# 

**Published:** 1892-09-17

**Authors:** 


CONTENTS.

| | PAGE |
|---|---|
| **Cholera Inoculation** | 401 |
| **Passing Topics.**—The Pleasantries of the Mob | 402 |
| **Cholera Precautions** | 403 |
| **Women as Workers.**—II. Where Women Need Not Fail | 404 |
| **Medical History from the Earliest Times.** By E. T. WITHINGTON, M.A., M.B.Oxon. XXIV.—The Influence of Christianity on Medicine | 405 |
| **Everybody's Page** | 406 |
| **Annotations.**—The Lourdes Miracles—Poorer Ireland in 1891—A Generous Nurse | 407 |
| **Notes and News** | 408 |
| **Scraps and Gleanings** | 409 |
| **The Practitioner's Mirror and Retrospect—** | |
| MEDICINE.—On Chronic Pharyngitis 410; Diabetes | 411 |
| SURGERY.—The Treatment of Internal Hæmorrhage | 411 |
| **Practitioner's Bookshelf.**—Cardiac Outlines | 412 |
| **The Institutional Workshop—** | |
| PRACTICAL DEPARTMENTS. II.—The Laundry | 413 |
| **Hospital Worthies.**—Mr. William John Nixon | 414 |
| **The Cure of Poverty.**—An Account of the North Kensington Friendly Workers among the Poor. By Mrs. C. FURLEY-SMITH | 415 |
| **The Student of Medicine.**—Appointments—Vacancies | |

**EXTRA SUPPLEMENT.—THE NURSING MIRROR.**

| | |
|---|---|
| **En Passant.**—A Trained Nurse for Sidcup—The Victoria Hospital for Children, Chelsea—Prince Alfred Hospital, Sydney—Withe ed Window Plants—A Pretty Picture—Short Items—Lady Lecturers—The Foresters and Salisbury Infirmary—Nurses' Recreation | clxxv |
| **How to Become a Medical Woman** | clxxvi |
| **Ladies' Conference at the Sanitary Congress** | clxxvii |
| **Our Australian Letter** | clxxvii |
| **Our Indian Letter** | clxxviii |
| **An Amusing Lecture** | clxxviii |
| **Death in Our Ranks** | clxxix |
| **Royal National Pension Fund for Nurses** | clxxix |
| **Appointment—Presentation** | clxxix |
| **Everybody's Opinion.**—General Hospital, Birmingham—Milk and Scarlet Fever—Holiday Homes for Northen Nurses, Scarborough | clxxix |
| **Off Duty.**—"The Result of a Chill." | clxxx |
| **Notes and Queries—Wants and Workers** | clxxx |

